# Glucocorticoids employ the monomeric glucocorticoid receptor to potentiate vitamin D_3_ and parathyroid hormone–induced osteoclastogenesis

**DOI:** 10.1096/fj.201802729RRR

**Published:** 2019-11-14

**Authors:** H. Herschel Conaway, Petra Henning, Antia Lie, Jan Tuckermann, Ulf H. Lerner

**Affiliations:** * Department of Physiology and Biophysics, University of Arkansas for Medical Sciences, Little Rock, Arkansas, USA;; † Center for Bone and Arthritis Research, Institute for Medicine, Sahlgrenska Academy at University of Gothenburg, Gothenburg, Sweden;; ‡ Department of Molecular Periodontology, Umeå University, Umeå, Sweden;; § Institute of Comparative Molecular Endocrinology, University of Ulm, Ulm, Germany

**Keywords:** osteoporosis, bone resorption, calcium-regulating hormones, osteoclasts

## Abstract

Glucocorticoid (GC) therapy decreases bone mass and increases the risk of fractures. We investigated interactions between the GC dexamethasone (DEX) and the bone resorptive agents 1,25(OH)_2_-vitamin D_3_ (D3) and parathyroid hormone (PTH) on osteoclastogenesis. We observed a synergistic potentiation of osteoclast progenitor cell differentiation and formation of osteoclasts when DEX was added to either D3- or PTH-treated mouse bone marrow cell (BMC) cultures. Cotreatment of DEX with D3 or PTH increased gene encoding calcitonin receptor (*Calcr*), acid phosphatase 5, tartrate resistant (*Acp5*), cathepsin K (*Ctsk*), and TNF superfamily member 11 (*Tnfsf11*) mRNA, receptor activator of NF-κB ligand protein (RANKL), numbers of osteoclasts on plastic, and pit formation and release of C-terminal fragment of type I collagen from cells cultured on bone slices. Enhanced RANKL protein expression caused by D3 and DEX was absent in BMC from mice in which the GC receptor (GR) was deleted in stromal cells/osteoblasts. Synergistic interactions between DEX and D3 on RANKL and osteoclast formation were present in BMC from mice with attenuated GR dimerization. These data demonstrate that the GR cooperates with D3 and PTH signaling, causing massive osteoclastogenesis, which may explain the rapid bone loss observed with high dosages of GC treatment.—Conaway, H. H., Henning, P., Lie, A., Tuckermann, J., Lerner, U. H. Glucocorticoids employ the monomeric glucocorticoid receptor to potentiate vitamin D_3_ and parathyroid hormone–induced osteoclastogenesis.

Glucocorticoids (GCs) are used clinically for their anti-inflammatory and immunosuppressive functions. The long-term use of GCs, or excess of endogenous GCs due to disease in the adrenal gland or because of enhanced ACTH stimulation from the pituitary gland, are associated with detrimental effects in many organs and tissues, including bone and muscles (1). A common side effect is decreased bone mineral density (BMD) with increased risk of fracture, initially described by Cushing (2) in 1932. In fact, GC-induced osteoporosis is the most common form of secondary osteoporosis (3). For reasons not understood, GC-induced fractures can occur when the loss of BMD has not been as profound as the loss normally associated with fractures in postmenopausal women with primary osteoporosis (4, 5). Fractures may occur in 20–50% of patients receiving chronic GC treatment (6–8), with the prevalence of fracture increasing with age and dose (8). Cortical bone loss induced by GCs is more pronounced in vertebra than in the forearm (9) and is associated with significantly more trabecular bone loss than in postmenopausal osteoporosis (10), which are reasons why GC-induced fractures are more common in sites with high amounts of trabecular bone, such as the vertebra and femoral neck (11).

Independent of the underlying disease, GC-induced bone loss results in an initial rapid loss of BMD and an increased fracture risk as early as 3–6 mo after initiation of therapy. A lower rate of bone loss is observed after 1 yr (11). The early phase of bone loss is caused by increased bone resorption, whereas the long-term loss is due to decreased bone formation (1, 3, 12–15). Decreased bone formation is caused by a direct effect on osteoblasts, resulting in decreased numbers and differentiation of osteoblasts (16). It is also suggested that increased osteoblast and osteocyte apoptosis play an important role in GC-induced decreased bone formation (16–19). The early phase of increased bone resorption has been attributed to increased formation (20–22) and survival (23) of osteoclasts, as well as to increased activity of mature osteoclasts (24, 25).

Osteoclasts are multinucleated cells derived from mononucleated progenitor cells in the myeloid cell lineage (26). The proliferation and survival of progenitor cells is dependent on M-CSF, and their differentiation and fusion to mature osteoclastsis is dependent on the receptor activator of NF-κB ligand (RANKL) (27). The amount of active RANKL is limited by the decoy receptor osteoprotegerin (OPG), which binds to RANKL. M-CSF is expressed in many different cell types, including osteoblasts, whereas the expression of RANKL is restricted mainly to osteoblasts, osteocytes, and stromal cells in the bone marrow. Downstream events subsequent to activation of the receptor RANK in osteoclast progenitor cells include activation of osteoclastogenic transcription factors such as NF-κB, c-Fos, and nuclear factor of activated T cells, cytoplasmic 1 (NFATc1), the latter being regarded as the master transcription factor for osteoclastogenesis (28). The transcription factors induce a wide variety of genes important for osteoclast differentiation and function, including calcitonin receptor (*Calcr*) and acid phosphatase 5, tartrate resistant (*Acp5;* TRAP), both well-recognized markers of mature osteoclasts, and cathepsin K (*Ctsk*), which is involved in degradation of bone extracellular matrix.

Studies using a wide variety of mouse and primary osteoblasts, human bone marrow stromal cells, and osteoblastic cell lines have demonstrated that GCs decrease TNF receptor superfamily, member 11b [*Tnfrsf11b*; mRNA (encoding OPG)] (20, 29–31) and OPG protein (30, 32). It has also been shown using these cells, that GCs can induce TNF superfamily member 11 (*Tnfsf11*) mRNA (encoding RANKL) expression (20, 30, 31). Similarly, GCs induce *Tnfsf11* mRNA and RANKL protein in *ex vivo* cultures of neonatal mouse calvarial bones (21). A decrease of OPG in serum, associated with increased serum levels of C-terminal fragment of type I collagen (CTX) has been reported as early as 24 h in patients treated with prednisolone (14). The fact that treatment with anti-RANKL antibodies can decrease bone loss and osteoclast formation in mice treated with prednisolone suggests a crucial role for RANKL in GC-induced osteoporosis (22).

In *ex vivo* experiments using neonatal mouse calvarial bones, we have observed that addition of dexamethasone (DEX) or hydrocortisone to parathyroid hormone (PTH) or 1,25(OH)_2_-vitamin D_3_ (D3) can synergistically potentiate calcium release and bone matrix degradation from parietal, cortical bone (33). Fractures at skeletal sites with proportionally large amounts of trabecular bone are more common in patients with GC-induced osteoporosis than fractures at sites with predominantly cortical bone (9–11). Osteoclasts resorbing trabecular bone are derived from progenitors in bone marrow, and although little is known about which factors control trafficking of progenitors to bone surfaces, recent studies have shown that the Gαi protein-coupled receptor EBI2, expressed by osteoclast progenitors, and oxysterol ligands, produced by osteoblasts, are important regulators of this process (34). We have focused the present investigation on how GCs affect osteoclastogenesis in bone marrow cell (BMC) cultures in the absence and presence of D3 and PTH. Because GCs can regulate cellular activities not only by forming a complex with dimeric GC receptors (GRs) but also by acting through monomeric GR on the genome as well (35), we also performed experiments using cells from GR*^dim^* mice that have a point mutation in the dimerizing interface (36).

## MATERIALS AND METHODS

### Materials

Mouse OPG fused to human IgG_1_ Fc (OPG/Fc chimera), recombinant mouse M-CSF, recombinant extracellular domain of mouse RANKL (Arg72-Asp316; 462-TR), recombinant RANK/TNFRSF11A (RANKL neutralizing soluble RANK), and the ELISA kits for mouse RANKL and mouse OPG were purchased from R&D Systems (Minneapolis, MN, USA). The kit for leukocyte acid phosphatase staining, Sigma 104 phosphatase substrate, Trizol, DEX, hydrocortisone, prednisolone, and RU38486 were from MilliporeSigma (Burlington, MA, USA. Bovine PTH 1–34 was from Bachem (Bubendorf, Switzerland); α-modification of minimum essential medium (α-MEM), and fetal calf serum were from Thermo Fisher Scientific (Waltham, MA, USA); Thermo Sequenase- II DYEnamic ET Terminator Cycle Sequencing Kit was from Amersham (Little Chalfont, United Kingdom); and oligonucleotide primers were from Thermo Fisher Scientific. HotStar Taq Polymerase Kit, QiaQuick PCR Purification Kit and RNeasy Mini Kit were from Qiagen (Hilden, Germany); DNA-Free and RNAqueous–4PCR Kit were obtained from Ambion (Austin, TX, USA); First-Strand cDNA Synthesis Kit and the PCR Core Kit were from Hoffmann-La Roche (Basel, Switzerland). Fluorescent-labeled probes (reporter fluorescent dye VIC at the 5′end and quencher fluorescent dye Tamra at the 3′end), TaqMan Universal PCR Master Mix, and the kits for real-time quantitative PCR were from Thermo Fisher Scientific; culture dishes, multiwell plates, and glass chamber slides were from Thermo Fisher Scientific; suspension culture dishes from Corning (Corning, NY, USA); bone slices and CrossLabs for Culture ELISA (CTX) were from Immunodiagnostic Systems (East Boldon, United Kingdom); and the mouse bone marrow stromal cell line ST-2 was from Riken BioResource Research Center Cell Bank (Tsukuba, Japan). D3 was a kind gift from Hoffmann-La Roche or purchased from MilliporeSigma. D3, DEX, hydrocortisone, prednisolone, and RU38486 were dissolved in ethanol. All other compounds were dissolved either in PBS or culture medium.

### Animals

CsA mice from our own inbred colony were used in most experiments if not otherwise stated. The *GR^dim^* mice and their corresponding wild-type mice have been previously described and were backcrossed to the FVB/N background as described in Rauch *et al*. (16). The *GR^flox^* and *GR^Runx2Cre^* mice were generated as previously described (16). Animal care and experiments were approved and conducted in accordance with accepted standards of humane animal care and use as deemed appropriate by the Animal Care and Use Committee of Umeå University.

### Osteoclast formation in BMC cultures

Femurs and tibiae from 5- to 7-wk-old male mice were dissected and cleaned from adhering tissues. BMCs were seeded in 48 or 96 multiwells (10^6^ cells/cm^2^), incubated overnight in α-MEM/10% FBS, and subsequently cultured in the same medium with or without hormones and test substances, with concentrations as indicated in legends to figures, for 7–9 d, with medium changed every third day. After this interval, the cells were fixed with acetone in citrate buffer/3% formaldehyde and stained for TRAP. TRAP-positive cells with 3 or more nuclei were considered osteoclasts, and the number of multinucleated osteoclasts was counted (TRAP^+^ MuOCL). In parallel experiments, cells were harvested for gene expression analyses.

### Pit formation

Bone slices (6 mm in diameter) were placed in 96 multiwell plates and 50 μl α-MEM/10% FBS was added, and bone slices were incubated for 15 min at 37°C. Then, 50 μl α-MEM/10% FBS containing 4 × 10^5^ BMC was added. After attachment overnight, identical medium with or without hormones and test substances, as indicated, was added and the cells incubated for 8 or 12 d. Media were changed every third day. At the end of the incubations, cells were stained for TRAP and the number of osteoclasts counted. Subsequently, cells were removed by sonication in 0.5 M ammonium hydroxide and bone slices stained for pits with Toluidine blue. Bone resorbing activity was assessed by analyzing culture medium for CTX with an ELISA kit by following the instructions provided by the supplier.

### Bone marrow stromal cell culture

BMCs (10^6^ cells/cm^2^) were seeded in 25-cm^2^ flasks in α-MEM/10% FBS and cultured for 2 wk with medium changed every 3 d. After the 2-wk period, cells attached to the bottom of the flasks were detached with trypsin and seeded (2 × 10^4^ cells/cm^2^) as bone marrow stromal cells in 48 multiwell plates and incubated with or without D3 and DEX. After 24 or 48 h, RNA was isolated for gene expression analyses. The ST-2 cells were seeded in 24 multiwell plates at a density of 2 × 10^4^ cells/cm^2^ and incubated in α-MEM/10% FBS overnight. Following incubation, medium with and without test substances was added and the cells incubated for 24 h for subsequent gene expression analyses.

### RNA isolation and first-strand cDNA synthesis

Total RNA from cells was extracted using the RNAqueous–4PCR Kit according to the manufacturer’s instructions. Samples were subsequently digested with DNA-free. Single-stranded cDNA was synthesized from 1 μg of total RNA using a First-Strand cDNA Synthesis Kit with avian myeloblastosis virus and oligo(dT)_15_ primers, according to the manufacturer’s protocol. To ensure that there was no genomic DNA in the samples, negative controls that did not contain avian myeloblastosis virus reverse transcription were included. Expression of mRNA was determined using real-time quantitative PCR.

### Real-time quantitative PCRs

Real-time quantitative PCR analyses were performed using the TaqMan Universal PCR Master Mix and a sequence detection system (ABI Prism 7900 HT Sequence Detection System and Software or the StepOnePlusReal-Time PCR System; Thermo Fisher Scientific). All genes were analyzed using primers and probes that have been previously specified (37).

### TRAP protein analysis

BMCs were isolated, as described above, and seeded in 24- or 48-well plates at a cell density of 10^6^ cells/cm^2^. The cells were allowed to settle overnight before medium was changed, then cultured for an additional 6 d, with a change of medium after 3 d. After the 6-d culture, cells were washed in PBS and lysed in Triton X-100 (0.2% in H_2_O). Following centrifugation, supernatant was collected and analyzed for TRAP activity using para-nitrophenyl phosphate as substrate at pH 4.9, in the presence of tartrate (0.17 M). The activity of the enzyme was assessed as the OD_405_ of liberated para-nitrophenol. Enzyme assays were performed under conditions in which the reactions were proportional to the amount of enzyme and reaction time.

### RANKL and OPG protein analyses

BMC were cultured in 24 multiwell plates for 6 or 12 d in the absence and presence of hormones and test substances. Measurements of OPG and RANKL protein synthesis were assessed by analyzing the levels of OPG and RANKL in BMC and in culture medium using commercially available ELISAs. At the end of the incubations, proteins were extracted using 0.2% Triton X-100 for 24 h at room temperature, and the BMC extracts and media were analyzed by ELISA according to the manufacturer’s protocol.

### Statistics

All statistical analyses were performed using 1-way ANOVA with Levene’s homogenicity test, and *post hoc* Bonferroni’s, or where appropriate, Dunnett’s T3 test or the independent samples Student’s *t* test (SPSS for Windows; Apache Software Foundation, Forest Hill, MD, USA). All experiments have been performed at least twice with comparable results, and all data are presented as the means ± sem.

## RESULTS

### Osteoclast differentiation, formation, and activity caused by D3 in BMC is synergistically potentiated by GC

**Figure 1:**
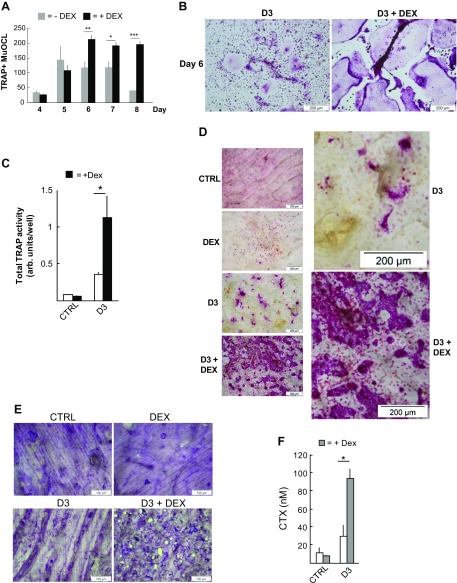
Potentiation of osteoclast formation induced by DEX+D3 in mouse BMC cultures. *A*–*C*) DEX (10^−7^ M) was added together with D3 (10^−8^ M) to mouse BMC cultures on plastic dishes. The number of TRAP^+^ multinucleated cells (TRAP^+^MuOCL) per well at d 4–8 (*A*). Images from TRAP-stained cells demonstrating the large size of TRAP^+^MuOCL in cells treated with DEX (*B*). *C*) Total TRAP activity in BMCs at d 6. BMCs were also incubated in control medium (ctrl) and in medium with DEX (10^−7^ M) and TRAP-stained; no TRAP^+^MuOCL cells were observed in the control medium, and only a few in cultures treated with DEX, unpublished results. *D*–*F*) Mouse BMCs were cultured on bone slices and incubated in ctrl, DEX (10^−7^ M), or in the presence of D3 (10^−8^ M) without (D3) or with DEX (D3+DEX) for 8 d. Representative images from cells stained with TRAP (*D*). Bone slices from which the cells have been cleaned off and slices then stained with Toluidine blue to visualize resorption pits (*E*). The amounts of CTX released to culture medium during the last 3 d. Values are means of 5–7 observations, and vertical bars represent se (*F*). *P < 0.05, **P < 0.01, ***P < 0.001.

Addition of D3 (10^−8^ M) to mouse BMCs resulted in enhanced formation of TRAP^+^MuOCL, which was maximal at d 5–7 and thereafter declined (**Fig. 1*A***). Treatment with DEX (10^−7^ M) alone marginally enhanced osteoclastogenesis, and no TRAP^+^MuOCL were observed in untreated controls (unpublished results). When DEX was added to D3-stimulated cultures, a synergistically enhanced number of TRAP^+^MuOCL was observed at d 6–8 (Fig. 1*A*). The TRAP^+^MuOCL at d 6 in the D3+DEX-stimulated cultures were more spread out and clearly larger than those in the D3-treated group (Fig. 1*B*). At d 8, many huge, oversized TRAP^+^MuOCL were seen in the D3+DEX group, whereas in the D3 group many apoptotic MuOCL were observed (Supplemental Fig. S1). The difference in size makes the counting of the total number of TRAP^+^MuOCL not fully appropriate to assess osteoclastogenesis. The 3-fold potentiation of intracellular Trap activity upon D3 and DEX exposure (Fig. 1*C*), which reflects the number of TRAP^+^ mononucleated osteoclast progenitors and multinucleated mature osteoclasts, suggests an important interaction between DEX and D3 to potentiate osteoclast differentiation in the BMC cultures.

The strong potentiation of TRAP^+^MuOCL formation was also observed when BMCs were incubated with D3 (10^−8^ M) and hydrocortisone (10^−6^ M) (Supplemental Fig. S2*A*). Similar to the observations for DEX, hydrocortisone cotreatment with D3 resulted in clearly larger TRAP^+^MuOCL (Supplemental Fig. S2*B*).

When BMCs were cultured on bone slices, a similar potentiation of TRAP^+^MuOCL formation was observed in cells cotreated with D3 (10^−8^ M) and DEX (10^−7^ M) (Fig. 1*D*, left). Although the morphology of the TRAP^+^MuOCL on bone slices was different from those on plastic, the combined treatment with DEX plus D3 also resulted in huge, oversized osteoclasts when BMCs were cultured on bone (Fig. 1*D*, right). These osteoclasts formed large islands, which made counting of numbers impossible. In unstimulated or DEX-stimulated BMCs on bone slices, only some few pits could be observed (Fig. 1*E*). In D3-stimulated BMCs, the number of pits was clearly enhanced, and in D3+DEX-stimulated cultures, a large number of pits was noted, some of which had even perforated the bone slices (Fig. 1*E*). In agreement with these observations, the release of CTX was unaffected by DEX itself but increased by D3, an effect synergistically potentiated in the presence of DEX (Fig. 1*F*).

### Expression of osteoclastic genes induced by D3 in BMC is synergistically potentiated by GC

**Figure 2:**
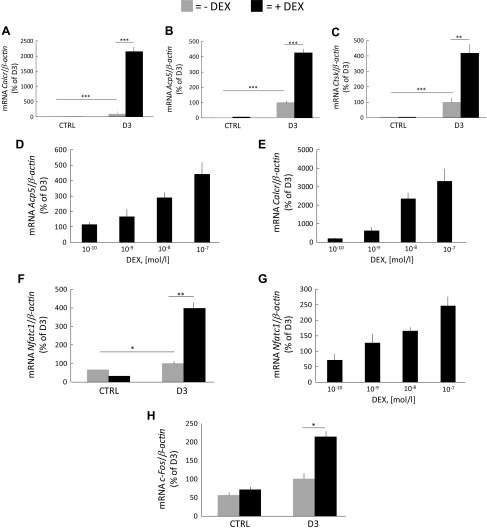
Potentiation of the mRNA expression of osteoclastic and osteoclastogenic genes induced by DEX plus D3 in mouse BMC cultures after 6 d of culture on plastic dishes. *A*–*C*) The mRNA expression of *Calcr* (*A*), *Acp5* (*B*), and *Ctsk* (*C*) in BMCs incubated in control medium (ctrl) with or without DEX (10^−7^ M) or in medium with D3 (10^−8^ M) with or without DEX. *D*, *E*) The effect by DEX at different concentrations when added together with D3 at 10^−8^ M on the mRNA expression of *Acp5* (*D*) and *Calcr* (*E*). *F*, *G*) The mRNA expression of *Nfatc1* in cells treated with D3 without or with DEX (10^−8^ M (*F*) or with DEX at different concentrations (*G*). *H*) The mRNA expression of *cFos* in cells treated with D3 without or with DEX (10^−8^ M). The mRNA expression of the genes was arbitrarily set to 100% in D3-stimulated cells in all figures. Values are means of 4 observations, and vertical bars represent se. *P < 0.05, **P < 0.01, ***P < 0.001.

To assess if stimulation of osteoclast formation was associated with increased differentiation of osteoclast progenitors in the BMC, rather than being caused by enhanced fusion, we analyzed expression of osteoclastic and osteoclastogenic genes. Cotreatment with D3 (10^−8^ M) and DEX (10^−7^ M) resulted in a robust synergistic enhancement at d 6 of *Calcr* mRNA (encoding calcitonin receptor), *Acp5* (encoding TRAP) and *Ctsk* (encoding cathepsin K) expression (**Fig. 2*A–C***). Up-regulations of these 3 osteoclastic genes were also observed at d 3, although to a lesser degree (unpublished results). The potentiation of *Calcr* mRNA expression by DEX was considerably larger (21.5-fold compared with D3 without DEX) than either that of *Acp5* or *Ctsk* (4.3- and 4.2-fold, respectively, compared with D3 without DEX). The synergistic up-regulation of the expression of *Calcr* and *Acp5* mRNA was concentration dependent with substantial effects at 10^−9^ M and higher concentrations of DEX (Fig. 2*D, E*).

The mRNA expression of the major osteoclastogenic transcription factor, *Nfatc1*, was cooperatively induced by DEX and D3 at d 6 (Fig. 2*F*), an effect dependent on the concentration of DEX (Fig. 2*G*). The mRNA expression of *c-Fos* was also drastically enhanced by cotreatment with D3 and DEX (Fig. 2*H*).

These data are presented in relation to the response obtained by D3; results are also shown as the relative expression of target gene to that of β-actin (Supplemental Fig. S3*A–E*).

These data indicate that the formation of oversized osteoclasts induced by GCs plus D3 in BMC is associated with enhanced differentiation of osteoclast progenitors and is not solely an effect of increased fusion.

### Enhanced RANKL is responsible for the synergism noted between DEX and D3

**Figure 3:**
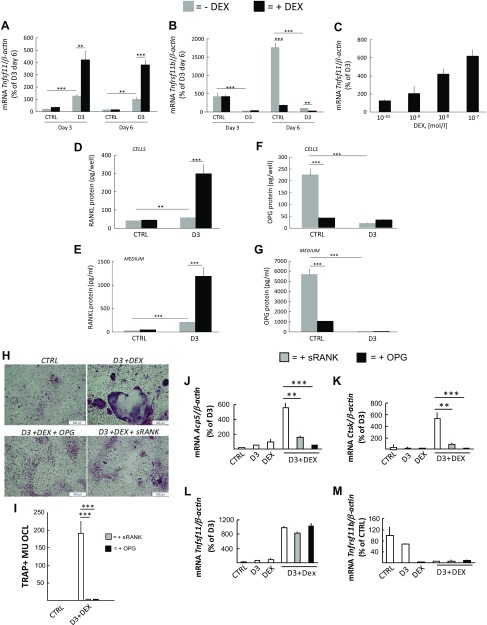
The synergistic potentiation of osteoclastogenesis in mouse BMC cultures treated with D3 plus DEX is due to synergistic potentiation of the mRNA and protein expression of RANKL stimulated by D3. *A*–*C*) The mRNA expression of *Tnfsf11* and *Tnfrsf11b* in BMCs at different time points and at different concentrations of DEX. The mRNA expression of *Tnfsf11* (*A*) and *Tnfrsf11b* (*B*) after 3 and 6 d in cells cultured in control medium (ctrl) with or without DEX (10^−7^ M) or in medium with D3 (10^−8^ M) with or without DEX. The effect of different concentrations of DEX addition on the mRNA expression of *Tnfsf11* at d 6 (*C*). The mRNA expression of genes in D3-stimulated cells at d 6 was arbitrarily set to 100%. *D*–*G*) The amount of RANKL and OPG protein in cell extracts and in culture medium (d 3–6) when BMCs were cultured in CTRL with or without DEX (10^−7^ M) or in medium with D3 (10^−8^ M)) with or without DEX for 6 d. *H*, *I*) BMCs were incubated with ctrl or medium containing D3 (10^−8^ M) and DEX (10^−7^ M) without or with soluble RANK (1 μg/ml) or OPG (300 ng/ml) for 6 d. *H*) Representative images of TRAP-stained cells. *I*) Number of TRAP^+^MuOCL per well. *J*–*M*) BMCs were incubated with ctrl or medium containing D3 (10^−8^ M) or DEX (10^−7^ M) or their combination (D3+DEX) without or with soluble RANK (1 μg/ml) or OPG (300 ng/ml) for 6 d. The mRNA expression of *Acp5* (*J*), *Ctsk* (*K*), *Tnfsf11* (*L*), and *Tnfrsf11b* (*M*) is shown relative to the expression in D3-stimulated cells. Values are means of 4 observations, and vertical bars represent se. **P < 0.01, ***P < 0.001.

The interaction between GCs and D3 in the BMC cultures may either occur at the level of RANKL- and OPG-expressing stromal cells, at the level of osteoclast progenitors, or may be due to effects mediated by other hematopoetic cells present. Therefore, it was evaluated if DEX affected D3 expression of RANKL and OPG. D3 (10^−8^ M) caused a 6-fold stimulation of *Tnfsf11* mRNA (encoding RANKL) in the BMC cultures at d 3 and 6 (**Fig. 3*A***). DEX (10^−7^ M) did not cause any effect by itself, but cotreatment with D3 and DEX caused robust, 19- and 24-fold enhancements of *Tnfsf11* mRNA at d 3 and 6, respectively. D3, and cotreatment with D3 and DEX, robustly decreased the time-dependent increase of *Tnfrsf11b* mRNA (encoding OPG) seen in control cultures over time (Fig. 3*B*). In separate experiments with BMC, it was found that cotreatment of DEX (10^−7^ M) with D3 caused a slight increase of *Tnfsf11* mRNA at 6 h and a strong induction at 24 h (Supplemental Fig. S4*A*). The expression of *Tnfrsf11b* mRNA was decreased by D3, an effect unaltered by cotreatment with DEX (Supplemental Fig. S4*B*). Synergistic up-regulation of *Tnfsf11* mRNA was concentration dependent and effective at 10^−9^ M DEX and higher concentrations (Fig. 3*C*). The strong inhibition (90%) of *Tnfrsf11b* mRNA expression induced by D3 was unaffected by all concentrations of DEX (unpublished results).

RANKL protein in cell lysates from BMC was slightly increased by D3 (10^−8^ M) 1.5-fold, but no effect by DEX (10^−7^ M) was observed (Fig. 3*D*). Cotreatment with D3+DEX resulted in a 7-fold increase of cellular RANKL protein. In medium, RANKL protein was enhanced by D3 7-fold and further increased by cotreatment with DEX (40-fold; Fig. 3*E*). OPG protein in cell lysates was clearly less abundant than in medium; both D3 and DEX substantially inhibited cellular OPG protein with no additional affect by cotreatment (Fig. 3*F*). OPG protein in medium was decreased 80% by DEX and by almost 100% by D3, with no further decrease by cotreatment (Fig. 3*G*).

The stimulatory effect by cotreatment with D3 and DEX (10^−7^ M) on TRAP^+^MuOCL formation was inhibited by OPG (300 ng/ml) and by soluble RANK (1 μg/ml) (Fig. 3*H, I*). OPG and soluble RANK abolished the enhanced mRNA expression of *Acp5* and *Ctsk* induced by D3+DEX (Fig. 3*J, K*) without affecting *Tnfsf11* or *Tnfrsf11b* mRNA expression (Fig. 3*L, M*).

The expression of colony-stimulating factor 1 (*Csf1*; encoding CSF1 or M-CSF) mRNA was strongly inhibited by D3 as well as by D3+DEX (Supplemental Fig. S4*C*).

The mRNA expression of *Nr3c1* (encoding GR) was not significantly regulated by D3, DEX, or by cotreatment with D3+DEX (Supplemental Fig. S5*A*). The vitamin D receptor (*Vdr*) was also not regulated by D3 and DEX nor by cotreatment with D3+DEX (Supplemental Fig. S5*B*).

### PTH-induced osteoclast differentiation, formation, and RANKL expression is potentiated by GC

**Figure 4:**
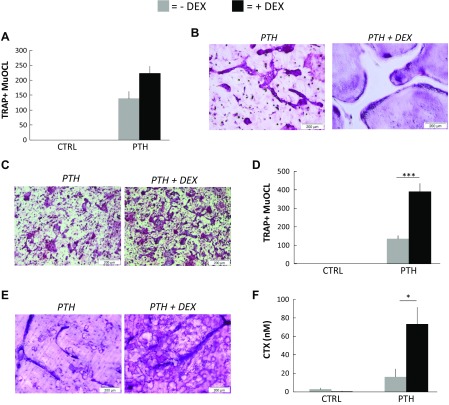
Potentiation of osteoclast formation in mouse BMC cultures treated with DEX and PTH. *A*, *B*) BMCs were incubated in control medium (ctrl) without or with DEX (10^−7^ M) or in medium with PTH (10^−8^ M) without or with DEX for 12 d on plastic dishes. The number of TRAP^+^MuOCL per well (*A*). Representative images of TRAP-stained BMCs (*B*). *C*–*F*) BMCs were cultured on bone slices for 12 d in the same medium as in *A* and *B*. Representative images of TRAP-stained bone slices (*C*). The number of TRAP^+^MuOCL per bone slice (*D*). Bone slices from which the cells have been cleaned off and slices then stained with Toluidine blue to visualize resorption pits (*E*). The amounts of CTX released to culture medium during the last 3 d (*F*). Values are means of 4 observations and vertical bars represent se. *P < 0.05, ***P < 0.001.

To investigate whether the interaction between D3 and GCs on RANKL and osteoclast formation in BMC was specific for these 2 hormones, we next assessed if DEX addition could potentiate PTH-induced osteoclast formation in the BMC cultures. PTH (10^−8^ M) stimulation of the number of TRAP^+^MuOCL formation was enhanced by costimulation with DEX (10^−7^ M) (**Fig. 4*A***), although the difference did not reach statistical significance. No TRAP^+^MuOCL was observed in unstimulated or DEX-stimulated cultures (unpublished results). The TRAP^+^MuOCL in PTH+DEX-treated cultures were substantially larger than those in PTH-stimulated BMC cultures (Fig. 4*B*), and, similar to observations in D3+DEX-stimulated BMC, many TRAP^+^MuOCL were very spread out, forming oversized TRAP^+^MuOCL in PTH+DEX-stimulated cultures as well. Therefore, counting the number of TRAP^+^MuOCL does not properly reflect the degree of osteoclast formation.

When BMCs were cultured on bovine slices, it was observed that DEX (10^−7^ M) plus PTH (10^−8^ M) further enhanced TRAP^+^MuOCL formation (Fig. 4*C, D*) in comparison with single PTH treatment. Similar to the observations in D3+DEX-stimulated BMC on bone (Fig. 1*D*), the TRAP^+^MuOCL in PTH+DEX-stimulated cultures formed contacts with each other in islands (Fig. 4*C*), although not to the same extent as in D3+DEX-stimulated cultures. Considerably more pits were formed in PTH+DEX-treated BMCs compared with single PTH treatment (Fig. 4*E*), which resulted in a very robust synergistic stimulation of CTX release (Fig. 4*F*).

**Figure 5:**
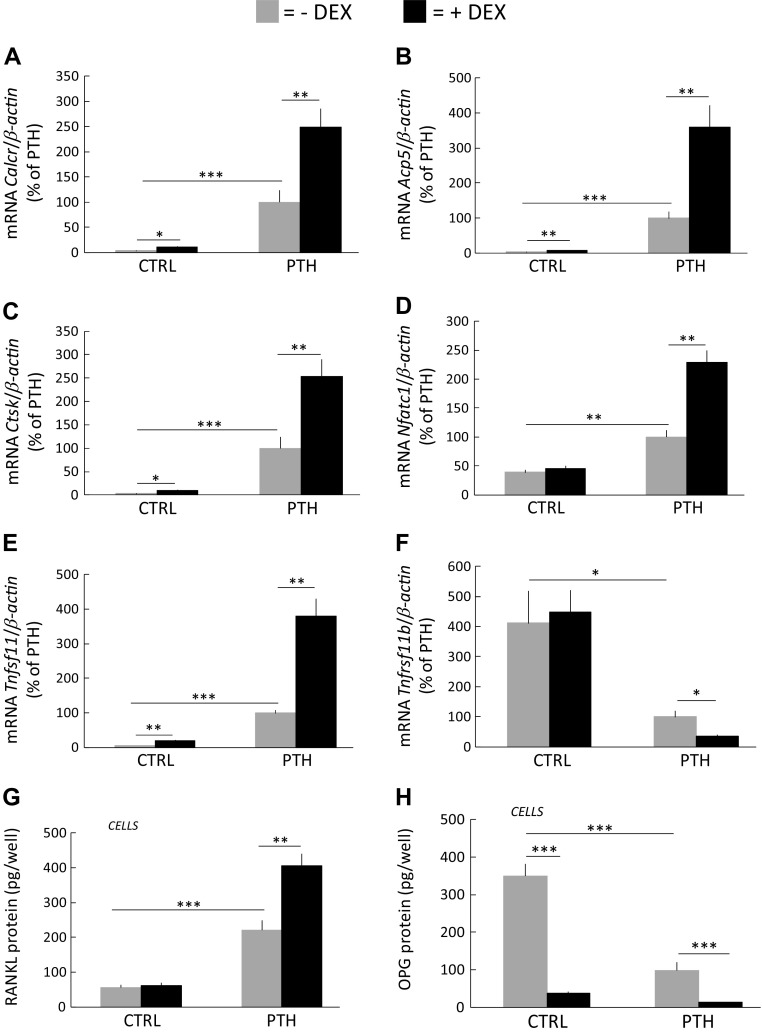
Potentiation of the mRNA expression of osteoclastic and osteoclastogenic genes and RANKL protein in mouse BMC cultures on plastic dishes treated with DEX and PTH. BMCs were incubated in control medium (ctrl) with or without DEX (10^−7^ M) or in medium with PTH (10^−8^ M) with or without DEX (D3). *A–D*) The mRNA expression at d 9 of *Calcr* (*A*), *Acp5* (*B*), *Ctsk* (*C*), and *Nfatc1* (*D*). *E*, *F*) The mRNA expression at d 6 of *Tnfsf11* (*E*) and *Tnfrsf11b* (*F*). *G*, *H*) The amounts of RANKL (*G*) and OPG (*H*) protein in cells at d 12. Values are means of 4 observations and vertical bars represent se. *P < 0.05, **P < 0.01, ***P < 0.001.

In line with these observations, PTH-induced mRNA expressions of *Calcr*, *Acp5*, *Ctsk*, and *Nfatc1* were synergistically potentiated in the presence of DEX (10^−7^ M) (**Fig. 5*A–D***).

PTH-enhanced *Tnfsf11* mRNA expression in BMC cultures was further increased upon DEX cotreatment (Fig. 5*E*). The mRNA expression of *Tnfrsf11b* in BMC cultures was decreased by PTH, and cotreatment further depressed the response (Fig. 5*F*).

Also, the protein levels of RANKL were enhanced by PTH alone and further potentiated in the presence of DEX (Fig. 5*G*), whereas OPG protein levels in BMC cells were decreased by DEX and PTH with further decreased levels seen by PTH and DEX cotreatment (Fig. 5*H*).

Intriguingly, the mRNA expression of *Nr3c1* (encoding GR) was not significantly regulated by PTH, DEX, or by cotreatment with PTH and DEX (Supplemental Fig. S5*C*). DEX, however, enhanced PTH receptor 1 (*Pthr1*) mRNA expression, but neither PTH nor PTH+DEX significantly affected *Pthr1* mRNA expression (Supplemental Fig. S5*D*).

### D3-induced RANKL expression in bone marrow stromal cells is synergistically potentiated by GC

These data demonstrate that cotreatment with GCs and either D3 or PTH results in a strong potentiation of osteoclast differentiation in BMC cultures due to an enhanced RANKL/OPG ratio, indicating that the interactions take place at the level of bone marrow stromal cells. We, therefore, purified stromal cells from mouse bone marrow and assessed the effects of cotreatment on *Tnfsf11* and *Tnfrsf11b* mRNA expression.

**Figure 6:**
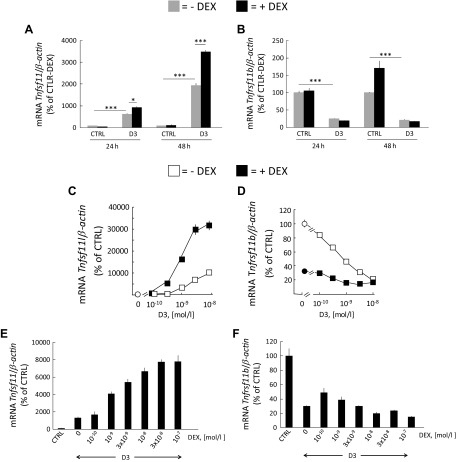
Potentiation of the mRNA expression of *Tnfsf11* in primary bone marrow stromal cells and in the ST-2 bone marrow stromal cell line treated with DEX and D3. *A*, *B*) Primary mouse bone marrow stromal cells were incubated with control medium (ctrl) without or with DEX (10^−7^ M) or with D3 (10^−8^ M) with or without DEX. The mRNA expression of *Tnfsf11* (*A*) and *Tnfrsf11b* (*B*) was analyzed after 24 and 48 h. *C*, *D*) ST-2 cells were incubated in either ctrl, medium with DEX (10^−7^ M), or different concentration of D3 with or without DEX (10^−7^ M). The mRNA expression of *Tnfsf11* (*C*) and *Tnfrsf11b* (*D*) was analyzed after 24 h. *E*, *F*) ST-2 cells were incubated ctrl or medium with D3 (10^−8^ M) without or with different concentrations of DEX. The mRNA expression of *Tnfsf11* (*E*) and *Tnfrsf11b* (*F*) was analyzed after 24 h. Values are means of 4 observations, and vertical bars represent se. *P < 0.05, ***P < 0.001.

A time-dependent increase in *Tnfsf11* mRNA expression was observed with D3 (10^−8^ M), which was potentiated by cotreatment with DEX (10^−7^ M) in purified primary bone marrow stromal cells (**Fig. 6*A***). In these cells, D3 substantially inhibited *Tnfrsf11b* mRNA expression at 24 and 48 h, a response unaffected by DEX (Fig. 6*B*).

Effects and interaction on *Tnfsf11* mRNA expression by D3 and DEX were also assessed in the mouse bone marrow stromal cell line ST-2. Treatment of the ST-2 cells with D3 (10^−10^–10^−7^ M) caused a robust, concentration-dependent stimulation of *Tnfsf11* mRNA (Fig. 6*C*), with effects noted at and above 3 × 10^−10^ M D3. Cotreatment of the ST-2 cells with DEX (10^−7^ M) synergistically potentiated *Tnfsf11* mRNA at all concentrations of D3. D3 inhibited *Tnfrsf11b* mRNA in a concentration-dependent manner at and above 10^−10^ M in the ST-2 cells (Fig. 6*D*). DEX (10^−7^ M) robustly decreased *Tnfrsf11b* mRNA and slightly enhanced the inhibitory effect by D3 at different concentrations (Fig. 6*D*).

The synergistic potentiation of *Tnfsf11* mRNA by DEX+D3 (10^−8^ M) in ST-2 cells was concentration dependent with the EC_50_ at ∼3 × 10^−9^ M DEX (Fig. 6*E*). The strong inhibition by D3 (10^−8^ M) on *Tnfrsf11b* mRNA was not further enhanced by DEX at different concentrations (Fig. 6*F*). Taken together, D3 and DEX treatment lead to a synergistic induction of *Tnfsf11/Tnfrsf11b* ratio in primary and immortalized stromal cells.

### Synergistic interaction by D3 and GC dependent on activation of GR and VDR

**Figure 7:**
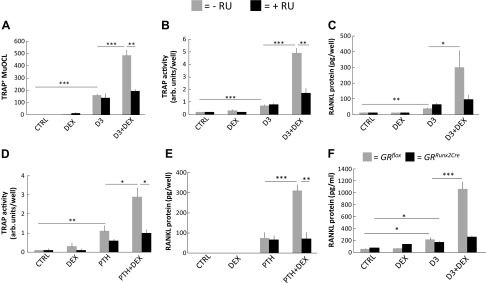
Potentiation of osteoclastogenesis and RANKL protein in mouse BMCs by the combination of DEX plus D3 or DEX plus PTH is dependent on GRs in *Runx2* expressing stromal cells. *A*–*C*) BMCs were incubated in either control medium (ctrl) without or with the GR antagonist RU 384 (10^−6^ M), medium containing DEX (10^−7^ M) without or with RU 384, medium containing D3 (10^−8^ M) without or with RU 384, and medium containing D3+DEX without or with RU 384. The number of TRAP^+^MuOCL per well after 7 d (*A*). The amount of cellular TRAP activity after 6 d (*B*). The amount RANKL protein in the cells after 6 d (*C*). *D*, *E*) BMCs were incubated either in ctrl without or with the GR antagonist RU 384 (10^−6^ M), in medium containing DEX (10^−7^ M) without or with RU 384, in medium containing PTH (10^−8^ M) without or with RU 384, or in medium containing PTH+DEX without or with RU 384 (*C*). Cellular TRAP activity after 12 d (*D*). The amount of RANKL protein in the cells after 12 d (*E*). *F*) BMCs from *GR^flox^* and *GR^Runx2Cre^* mice were incubated in either CTRL, medium containing DEX (10^−7^ M), medium containing D3 (10^−8^ M), or medium containing D3+DEX and RANKL protein in the cells analyzed after 6 d. Values are means of 4 observations, and vertical bars represent se. *P < 0.05, **P < 0.01, ***P < 0.001.

The potentiation by DEX (10^−7^ M) on D3 (10^−8^ M) that stimulated formation of TRAP^+^MuOCL in BMC cultures was abolished by the GR antagonist RU 384 (10^−6^ M), with no effect observed by the antagonist on TRAP^+^MuOCL formation induced by only D3 (**Fig. 7*A***). In a parallel experiment, it was found that RU 384 also inhibited the interaction between D3 and DEX on cellular TRAP activity in BMC cultures (Fig. 7*B*). Addition of RU 384 to BMC did not affect D3-stimulated RANKL protein but abolished the synergistic interaction with DEX (10^−7^ M) (Fig. 7*C*).

Similarly, the stimulation of cellular TRAP activity and RANKL protein induced by cotreatment with PTH (10^−8^ M) and DEX (10^−7^ M) was abolished by RU 384 (Fig. 7*D, E*).

These data indicate that the potentiation of D3- and PTH-induced osteoclastogenesis and RANKL formation in the presence of DEX is dependent on GR, most likely GR in stromal cells. To specifically address the role of GR in stromal cells, we compared the effects on RANKL formation by D3 ± DEX in BMC cultures from *GR^flox^* and *GR^Runx2Cre^* mice. Robust synergistic potentiation by DEX+D3 of RANKL protein was observed in BMC from *GR^flox^* but not from *GR^Runx2Cre^* mice with a deletion of GR in osteoblast progenitors and descendent cells (16) (Fig. 7*F*).

These data show that the synergistic interaction between DEX and either D3 or PTH on RANKL formation and osteoclastogenesis is dependent on the receptors for GCs and that it is the GRs in stromal cells, which are important.

### Synergistic potentiation of osteoclast formation by GCs occurs in the absence of GR dimerization

**Figure 8:**
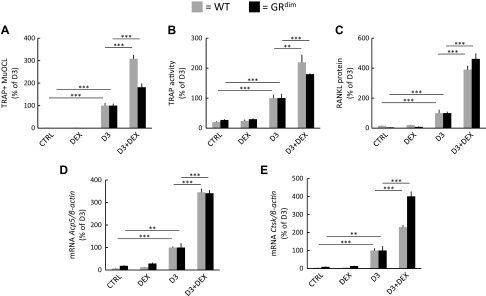
Potentiation of osteoclastogenesis, RANKL protein, and osteoclastic genes in mouse BMCs treated with DEX and D3 is not dependent on GR dimerization. *A*–*E*) BMCs were isolated from wild-type mice (WT) or from *GR^dim^* mice, carrying a mutation in the GR causing inhibition of receptor dimerization, and were incubated in either control medium (ctrl), medium with DEX (10^−7^ M), D3 (10^−8^ M), or D3+DEX. *A*) The number of TRAP^+^MuOCL per well. *B*) Cellular TRAP activity after 7 d. *C*) RANKL protein in the cells after 6 d. *D*, *E*) The mRNA expression of *Acp5* (*D*) and *Ctsk* (*E*) after 7 d. Values are means of 4 observations, and vertical bars represent se. **P < 0.01, ***P < 0.001.

To assess whether GR dimerization is required for the up-regulation of osteoclastogenesis, we performed experiments using cells from mice with disruption of one of the GR dimerizing interfaces, the d-box of the ligand-binding domain (38). Synergistic stimulation of TRAP^+^MuOCL formation in BMCs treated with D3 and DEX was observed in cultures from both *GR^dim^* and corresponding wild-type mice (**Fig. 8*A***). Similarly, synergistic stimulation of total TRAP activity (Fig. 8*B*) and RANKL protein (Fig. 8*C*) was found in D3- and DEX-stimulated BMCs from both *GR^dim^* and the wild-type mice. This is in contrast to up-regulation of the GR dimer-dependent target gene *Gilz* by DEX, which was not further enhanced by cotreatment with D3 and DEX (Supplemental Fig. S6). In agreement with these data, synergistic stimulation of *Ctsk* and *Acp5* mRNA by the combination of D3 and DEX was seen in both *GR^dim^* and wild-type mice (Fig. 8*D, E*). The differences noted between wild-type and *GR^dim^* cultures in the degree of D3+DEX induction of osteoclast number and *Ctsk* mRNA does not entirely exclude a role of GR dimerization. Nonetheless, when GR dimerization is impaired, D3+DEX synergistically enhances osteoclastogenesis in comparison with D3 treatment alone.

## DISCUSSION

Chronic treatment with GCs is associated with decreased bone mass, secondary osteoporosis, and an increased risk for skeletal fractures soon after the onset of treatment. The early loss of bone mass is partially explained by increased bone resorption, whereas the long-term effects are mainly attributed to decreased bone formation (39). The enhanced early bone resorption has been suggested to be due to decreased OPG and increased RANKL stimulated by GCs (20, 21, 29–32). Because fractures at skeletal sites with proportionally large amounts of trabecular bone are more common in patients with GC-induced osteoporosis than fractures at sites with predominantly cortical bone (10), we have focused the present investigation on effects of GCs on BMCs. In this study we have found that: *1*) GCs do not induce osteoclast formation in BMC cultures but synergistically interact with D3 and PTH to potentiate osteoclast formation, *2*) GCs interact with D3 and PTH to increase mRNA and protein expression of RANKL in bone stromal cell, and *3*) the effects of GCs are due to GRs present in bone marrow stromal cells expressing *Runx2*, which act through GR homodimers.

We initially observed that DEX did not stimulate osteoclast formation in BMC cultures performed either on plastic dishes or on bone slices. This was surprising because GCs have been described to increase the RANKL/OPG ratio by decreasing OPG (20, 29–32) and increasing RANKL (20, 21, 30, 31) in different bone cell cultures, as well as decreasing OPG and increasing CTX in serum of patients treated with prednisolone (14). In agreement with these observations, we observed that DEX decreased OPG at both the mRNA and protein levels in BMC cultures, but we failed to find any effect on RANKL mRNA or protein. Although the decrease of OPG resulted in an increased RANKL/OPG ratio, this change was not sufficient to induce osteoclast formation in these BMC cultures treated only with DEX. When we stimulated osteoclast formation in the BMC cultures with either D3 or PTH, with or without GCs, we observed a robust synergistic potentiation of osteoclast formation in the presence of either DEX or hydrocortisone. The osteoclasts observed in the BMC cultures performed on plastic dishes and cotreated with DEX and either D3 or PTH were oversized and formed large pancake-like osteoclasts. This made counting of the number of osteoclasts difficult, such that subsequent osteoclast numbers did not fully reflect osteoclastogenesis. The formation of oversized osteoclasts has also been observed when pure bone marrow macrophages have been cotreated with RANKL and GCs in cell cultures performed in plastic dishes. These oversized osteoclasts were found to be dependent on GRs expressed in myeloid cells (25). Therefore, the oversized osteoclasts in this study are most likely due to an effect caused by a direct interaction with GCs on GRs present in the osteoclast progenitors present in BMC.

Importantly, the synergistic interaction between either D3 or PTH and DEX on osteoclast formation was also observed when the BMC cultures were performed on bone slices. In both D3- and PTH-stimulated BMC, cotreatment with DEX resulted in excessive formation of resorption pits and synergistic potentiation of the release of CTX from the bone slices. Similarly, we have previously observed that addition of GCs to D3 or PTH synergistically potentiate bone resorption *ex vivo* in neonatal mouse calvarial bones (33).

An explanation that could be posited for the stimulatory effect by DEX is that the GC had an effect on osteoclast survival by inhibiting osteoclast apoptosis (23, 40). GCs have been reported to inhibit osteoclast apoptosis, but we have recently reported that this effect can only be seen when osteoclasts are incubated on plastic surfaces and not when osteoclasts are incubated on bone slices (25). Because we also observed a robust potentiation of osteoclast formation in DEX+D3- and PTH-stimulated BMC cultures on bone slices, it is not likely that the effect is due to osteoclast survival. This is further corroborated by the potentiated up-regulation of *Acp5*, *Ctsk*, and *Calcr* as well as the osteoclastogenic transcription factors, *Nfatc1* and *cFos*, by D3 and PTH in the presence of DEX.

Because GRs, as well as the receptors for D3 and PTH, are present in osteoblasts and bone marrow stromal cells, it was evaluated if the synergistic effects were due to interactions in these cells. It was observed that synergistic potentiations of the mRNA expression of *Tnfsf11* and RANKL protein occurred when DEX was added to D3- or PTH-treated BMC cultures. As expected, D3 and DEX both decreased OPG mRNA and protein expression. These findings demonstrate that the stimulatory effects on the RANKL/OPG ratio by D3 and PTH are strongly up-regulated in the presence of DEX. The fact that addition of either soluble RANK or OPG abolished the synergistic stimulation of osteoclast formation and osteoclastic genes induced by cotreatment with D3 and DEX demonstrates the crucial role of the interaction between DEX and D3 in bone marrow stromal cells on RANKL formation.

As early as the 1980s, there had been observations showing that osteoclast formation in BMC cultures induced by D3 or PTH was occurring in close contact with islands of stromal cells, indicating that cell-to-cell interactions were important (41). This was later confirmed by the discovery of membrane-bound RANKL on stromal cells activating RANK on osteoclast progenitor cells (42, 43). Since then, it has been found that RANKL can also be present in a soluble form because of cleavage by proteolytic enzymes (44, 45). The soluble form can be detected in serum and found to be increased in certain diseases, such as rheumatoid arthritis and periodontitis (46, 47). By using a mouse in which the known cleavage sites of RANKL had been mutated, it was recently reported that soluble RANKL is not necessary for osteoclast formation and bone loss in ovariectomized mice (48). The lack of soluble RANKL did not affect bone mass in growing mice but did decrease osteoclast number and increased bone mass in adult mice. In our BMC experiments, particularly in cells co-stimulated with D3 and DEX, we observed that a large proportion of RANKL protein was secreted to the medium, but we do not know if the protein detected was a mature form of RANKL or proteolytically shedded RANKL, or if osteoclastogenesis in these BMC cultures is dependent on cellular or secreted RANKL.

We found a synergistic potentiation of *Tnfsf11* mRNA expression in primary bone marrow stromal cells and in immortalized ST-2 cells. This response was dependent on the GR as demonstrated by the abrogation of osteoclast formation and RANKL production by RU486 treatment (49) and the lack of potentiation on RANKL production using BMC from *GR^Runx2cre^* mice lacking the GR in the osteoblastic lineage (16). These findings suggest that VDR and GR cooperatively act to induce the *Tnfsf11* gene.

VDR and GR responsive motifs are present in the promoter region of the *Tnfsf11* gene. Using deletion mutants of mouse *Tnfsf11* promoters linked to luciferase reporters, it has been shown that specific domains in the promoter are necessary to activate this construct by D3 and DEX, respectively (50, 51), but how GCs and D3 act in concert to regulate the *Tnfsf11* promoter remains to be shown. In particular, our finding that the synergistic interaction between D3 and DEX still can be seen in BMC expressing a GR with impaired GR dimerization (*GR^dim^*) indicates that monomeric GR action is mediating this effect, either by binding to GRE half-sites (52) or by tethering to other transcription factors (53, 54). In contrast, we recently showed that the stimulatory effect by GCs on osteoclast progenitor cells, which subsequently will lead to an increased bone resorbing activity in mature osteoclasts, is dependent on GR dimerization (25). Previously, we have reported that the GC-stimulated bone resorption in *ex vivo* cultures of mouse calvarial bones (33) is dependent on increased RANKL formation (21), and that stimulation of *Tnfsf11* mRNA is also seen in *GR^dim^* mice (55).

However, our observations demonstrate that GCs act in osteoblasts and bone marrow stromal cells to regulate RANKL formation through the monomeric receptor, whereas the effect in osteoclasts to enhance bone resorption is due to activation of the dimeric receptor.

**Figure 9:**
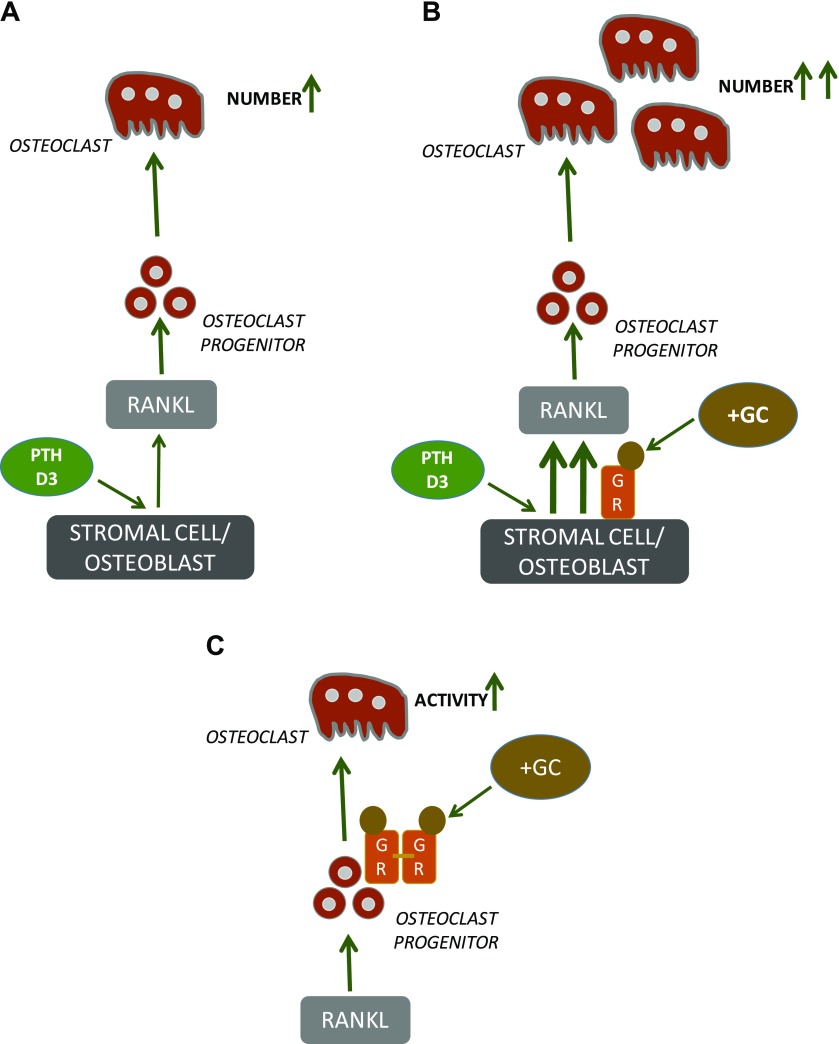
GCs can increase osteoclast formation and bone loss through 2 mechanisms: one by activation of GRs in bone marrow stromal cells and periosteal osteoblasts and the other is dependent on activation of GR in osteoclast progenitors. *A*) PTH and D3 increase osteoclast numbers by increasing RANKL in both stromal cells and periosteal osteoblasts and by decreasing OPG (not shown) in both cell types. GCs also cause a decrease of OPG in stromal cells, as shown in several studies, including the present study, but this results in only a marginal effect on the formation of multinucleated TRAP^+^ cells in BMC cultures (Results and Fig. 1). In calvarial osteoblasts, however, GCs increase both RANKL and, to a smaller extent, OPG, which results in activation of osteoclast differentiation and bone resorption in mouse calvarial bones (21, 33). *B*) Activation of GRs in bone marrow stromal cell cultures periosteal calvarial osteoblasts (21), which are concomitantly activated by either PTH or D3, results in synergistic up-regulation of RANKL (21) and robust formation of osteoclasts increased bone resorption (33); the synergistic effects in bone marrow stromal cells and periosteal osteoblasts are both dependent on monomeric GRs (55). *C*) Activation of GRs in osteoclast progenitor cells stimulated by RANKL does not affect the number of osteoclasts formed but does increase their bone resorbing activity; this effect is dependent on dimeric GRs (25).

Observations made by us and others in different *in vitro* and *ex vivo* experiments show that GCs can act at least by 3 different mechanisms to stimulate osteoclast formation and bone resorption: *1*) stimulating RANKL and decreasing OPG formation, *2*) synergistically interacting with D3 and PTH to potentiate RANKL formation, and *3*) enhancing the bone resorbing effect of mature osteoclasts. In **Fig. 9**, we depict how GCs can act on stromal cells/osteoblasts through the monomeric GR, and on osteoclast progenitors through the dimeric GR, to stimulate osteoclast number and activity.

In summary, we found that osteoclast formation was potentiated when GCs were added with either D3 or PTH to BMC cultures and that this stimulation is due to synergistic potentiation of RANKL formation and dependent on activation of monomeric GRs in bone marrow stromal cells.

## Supplementary Material

This article includes supplemental data. Please visit *http://www.fasebj.org* to obtain this information.

## Abbreviations

α-MEM: α–modification of minimum essential medium
*Acp5*: acid phosphatase 5, tartrate resistant
BMC: bone marrow cell
BMD: bone mineral density
*Calcr*: calcitonin receptor
*Ctsk*: cathepsin K
CTX: C-terminal fragment of type I collagen
D3: 1,25(OH)_2_-vitamin D3
DEX: dexamethasone
GC: glucocorticoid
GR: GC receptor
NFATc1: nuclear factor of activated T cells, cytoplasmic 1
OPG: osteoprotegerin
PTH: parathyroid hormone
RANKL: receptor activator of NF-κB ligand
*Tnfsf11*: TNF superfamily member 11
*Tnfrsf11b*: TNF receptor superfamily, member 11b
TRAP: tartrate-resistant acid phosphatase
MuOCL: multinucleated osteoclast
*Vdr*: vitamin D receptor
