# Supplementary material for: Glucocorticoids employ the monomeric glucocorticoid receptor to potentiate vitamin D3 and parathyroid hormone–induced osteoclastogenesis
Source: FASEB J. 2019 Nov 14;33(12):14394–409. doi: 10.1096/fj.201802729RRR (PMC6894088; doi:10.1096/fj.201802729RRR)
Supplement: Supplementary file 1 [file fj.201802729RRR.sd1.pdf]

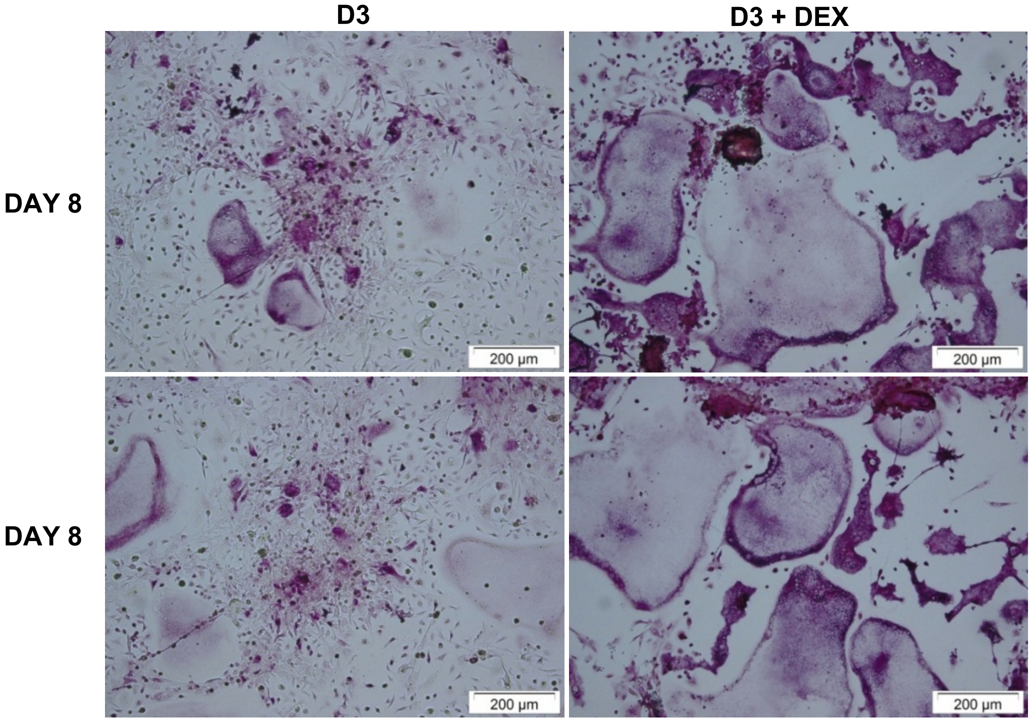

Supplemental figure 1. Co-treatment of mouse bone marrow cells (BMC) with 1,25$(OH)_2$-vitamin D3 (D3; 10^{-8}M) and dexamethasone (DEX; 10^{-7}M) for eight days results in formation of huge, oversized osteoclasts compared to treatment with D3 alone. In the D3 treated BMC several large apoptotic osteoclasts were seen.

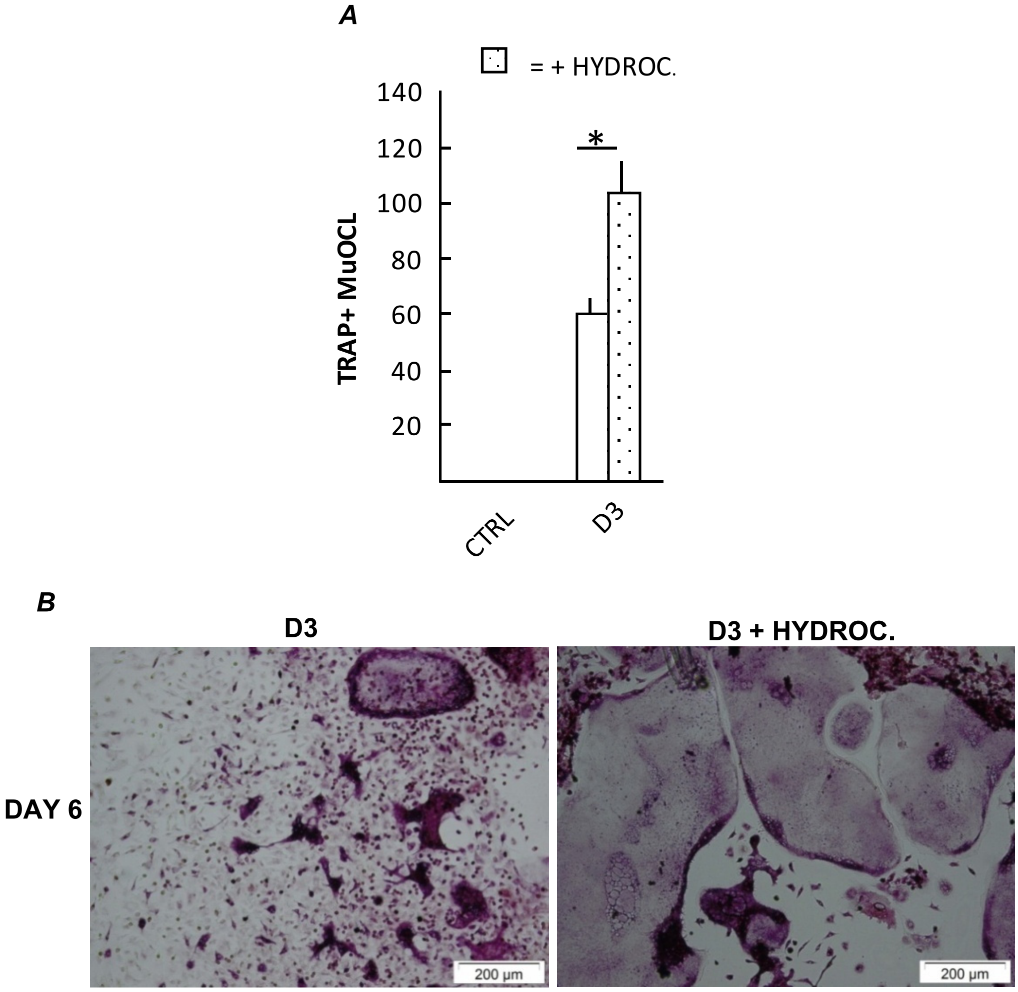

Supplemental figure 2. Hydrocortisone potentiates osteoclast formation induced by 1,25$(OH)_2$-vitamin D3 in mouse bone marrow cell (BMC) cultures. BMCs were incubated in control medium without or with hydrocortisone (HYDROC; 10^{-6}M) or with 1,25$(OH)_2$-vitamin D3 (D3; 10^{-8}M) without or with HYDROC. for seven days. *A*) Number of $TRAP^+$ multinucleated cells ($TRAP^+$MuOCL) per well. *B*) Representative images of TRAP-stained cells demonstrating the huge, oversized osteoclasts observed in hydrocortisone treated cells. *) $P<0.05$

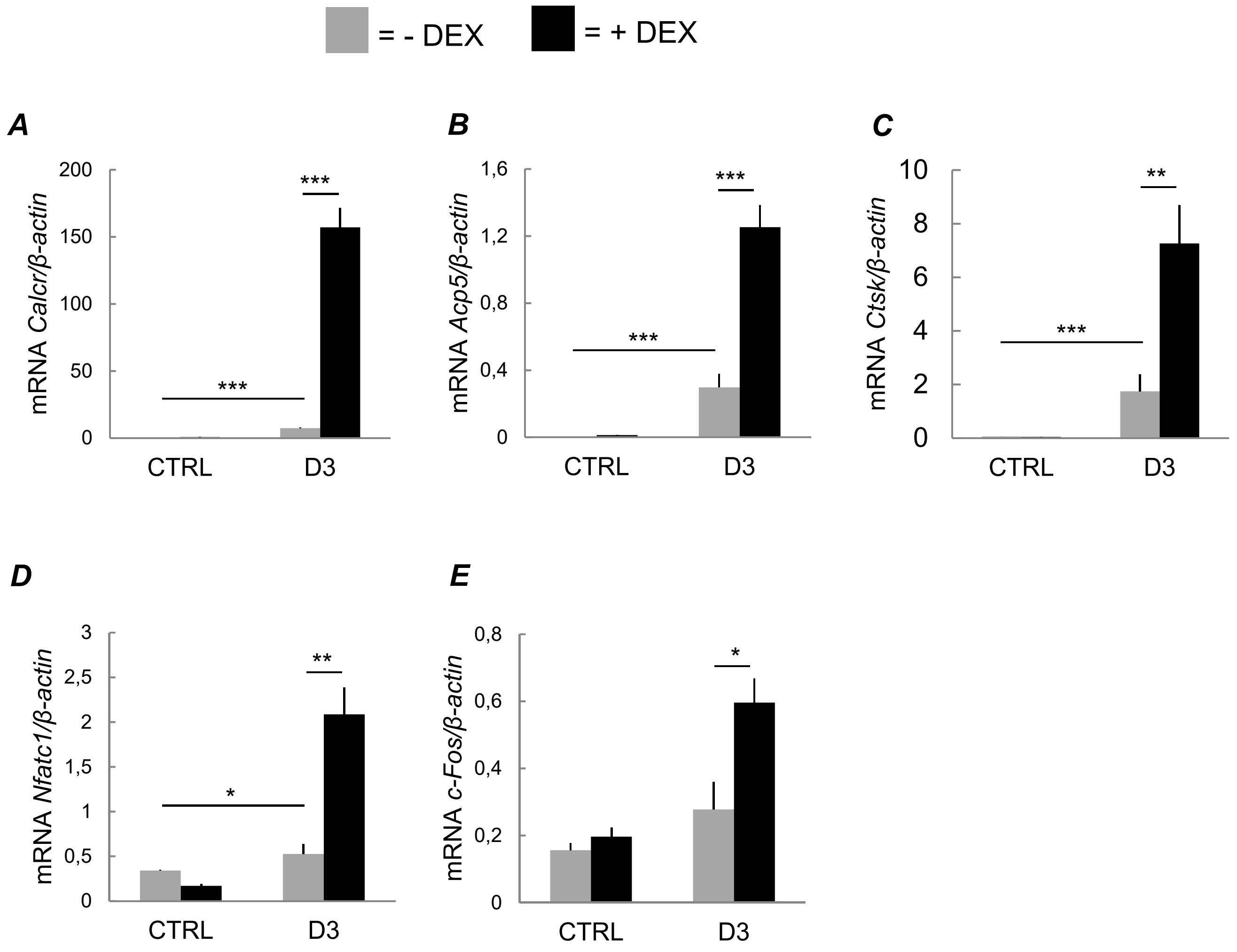

Supplemental figure 3. mRNA expression of osteoclastic and osteoclastogenic genes induced by dexamethasone plus 1,25(OH)$_2$-vitamin D3 in mouse bone marrow cell (BMC) cultures after six days of culture on plastic dishes. The mRNA expression of *Calcr* (*A*), *Acp5* (*B*), *Ctsk* (*C), Nfatc1* (D) and *c-Fos* (E) in BMC incubated in control medium with or without dexamethasone (DEX, 10^{-7}M; CTRL) or in medium with 1,25(OH)$_2$-vitamin D3 (10^{-8}M) with or without DEX (D3). Original qPCR data are the same as in main figure 2 but here expressed as mRNA /β-actin expression. Values are means of 4 observations and vertical bars represent SE. **P*<0.05, ***P*<0.01, ****P*<0.001.

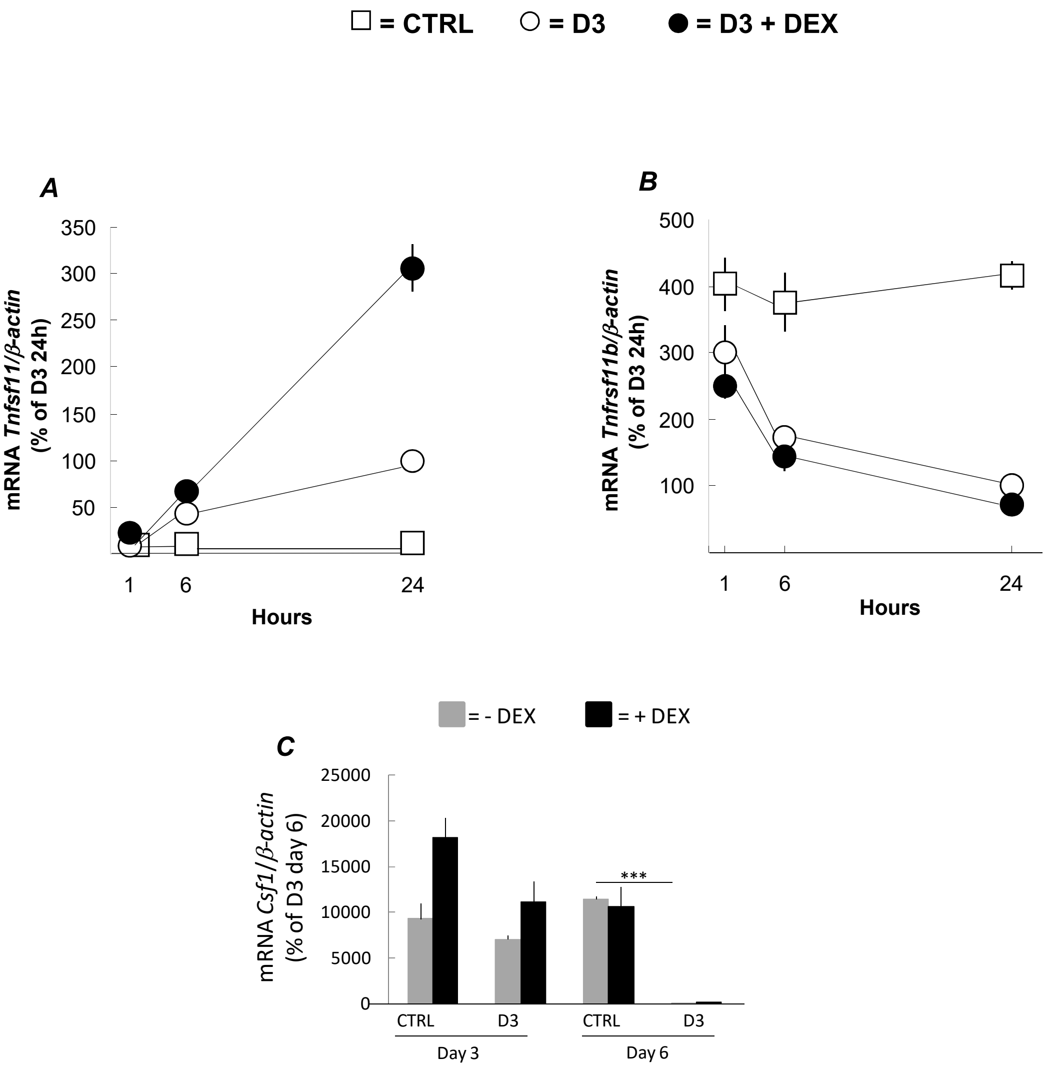

Supplemental figure 4. The effect by dexamethasone on the mRNA expression of RANKL, OPG and CSF1/M-CSF in mouse bone marrow cell (BMC) cultures stimulated by 1,25$(OH)_2$-vitamin D3. BMC were incubated in presence of dexamethasone (DEX; 10^{-7}M), 1,25$(OH)_2$-vitamin D3 (D3; 10^{-8}M) or in their combination. A) The mRNA expression of *Tnfsf11* at different time points. *B*) The mRNA expression of *Tnfrsf11b* at different time points. *C*) The mRNA expression of *Csf1* after three and six days in culture. ***) $P<0.001$.

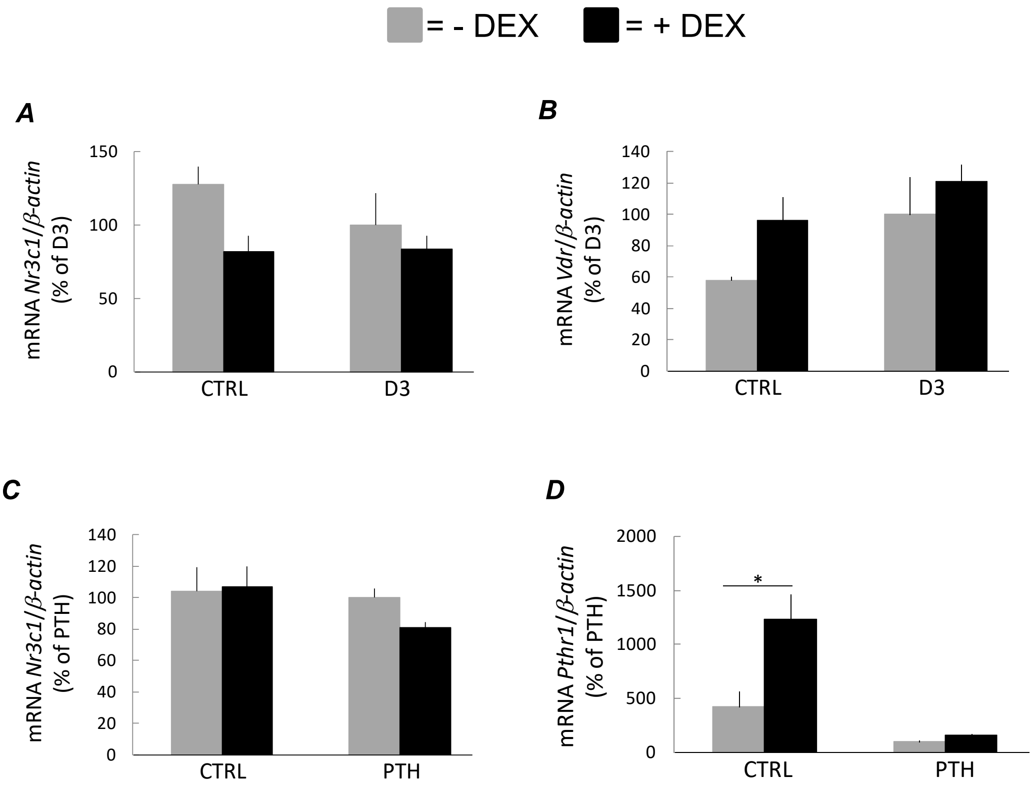

Supplemental figure 5. Lack of effect by dexamethasone on the mRNA expression of the receptors for glucocorticoids, vitamin D3 and parathyroid hormone in mouse bone marrow cell (BMC) cultures. A-D) BMCs were incubated in control medium without or with dexamethasone (DEX; 10^{-7}M) or in medium with either 1,25$(OH)_2$-vitamin D3 (D3; 10^{-8}M) or parathyroid hormone (PTH; 10^{-8}M) for six days. *A*) The mRNA expression of *Nr3c1* in D3 stimulated BMCs. *B*) The mRNA expression *Nr3c1* in D3 stimulated BMCs. *C*) The mRNA expression of *Vdr* in D3 stimulated BMCs. *D*) The mRNA expression of *Pthr1* in PTH stimulated BMCs. *) $P<0.05$

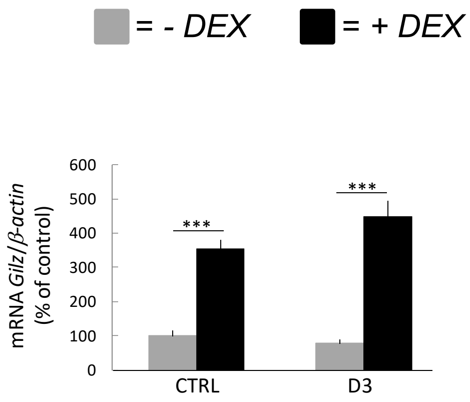

Supplemental figure 6. Lack of synergistic interaction between dexamethasone and vitamin D3 in mouse bone marrow cell (BMC) cultures from wild type mice on the mRNA expression of *Gilz* (glucocorticoid-induced leucine zipper). BMCs were incubated in control medium with or without dexamethasone (DEX; 10^{-7}M) or in medium with 1,25$(OH)_2$-vitamin D3 (D3; 10^{-8}M) with or without dexamethasone (DEX; 10^{-7}M) for six days. ***) $P<0.001$
